# Gender inequalities in the workplace: the effects of organizational structures, processes, practices, and decision makers’ sexism

**DOI:** 10.3389/fpsyg.2015.01400

**Published:** 2015-09-16

**Authors:** Cailin S. Stamarski, Leanne S. Son Hing

**Affiliations:** Department of Psychology, University of Guelph, GuelphON, Canada

**Keywords:** hostile sexism, benevolent sexism, institutional discrimination, human resources practices, gender harassment, personal discrimination

## Abstract

Gender inequality in organizations is a complex phenomenon that can be seen in organizational structures, processes, and practices. For women, some of the most harmful gender inequalities are enacted within human resources (HRs) practices. This is because HR practices (i.e., policies, decision-making, and their enactment) affect the hiring, training, pay, and promotion of women. We propose a model of gender discrimination in HR that emphasizes the reciprocal nature of gender inequalities within organizations. We suggest that gender discrimination in HR-related decision-making and in the enactment of HR practices stems from gender inequalities in broader organizational structures, processes, and practices. This includes leadership, structure, strategy, culture, organizational climate, as well as HR policies. In addition, organizational decision makers’ levels of sexism can affect their likelihood of making gender biased HR-related decisions and/or behaving in a sexist manner while enacting HR practices. Importantly, institutional discrimination in organizational structures, processes, and practices play a pre-eminent role because not only do they affect HR practices, they also provide a socializing context for organizational decision makers’ levels of hostile and benevolent sexism. Although we portray gender inequality as a self-reinforcing system that can perpetuate discrimination, important levers for reducing discrimination are identified.

## Introduction

The workplace has sometimes been referred to as an inhospitable place for women due to the multiple forms of gender inequalities present (e.g., Abrams, 1991). Some examples of how workplace discrimination negatively affects women’s earnings and opportunities are the gender wage gap (e.g., Peterson and Morgan, 1995), the dearth of women in leadership (Eagly and Carli, 2007), and the longer time required for women (vs. men) to advance in their careers (Blau and DeVaro, 2007). In other words, workplace discrimination contributes to women’s lower socio-economic status. Importantly, such discrimination against women largely can be attributed to human resources (HR) policies and HR-related decision-making. Furthermore, when employees interact with organizational decision makers during HR practices, or when they are told the outcomes of HR-related decisions, they may experience personal discrimination in the form of sexist comments. Both the objective disadvantages of lower pay, status, and opportunities at work, and the subjective experiences of being stigmatized, affect women’s psychological and physical stress, mental and physical health (Goldenhar et al., 1998; Adler et al., 2000; Schmader et al., 2008; Borrel et al., 2010),job satisfaction and organizational commitment (Hicks-Clarke and Iles, 2000), and ultimately, their performance (Cohen-Charash and Spector, 2001).

**FIGURE 1:**
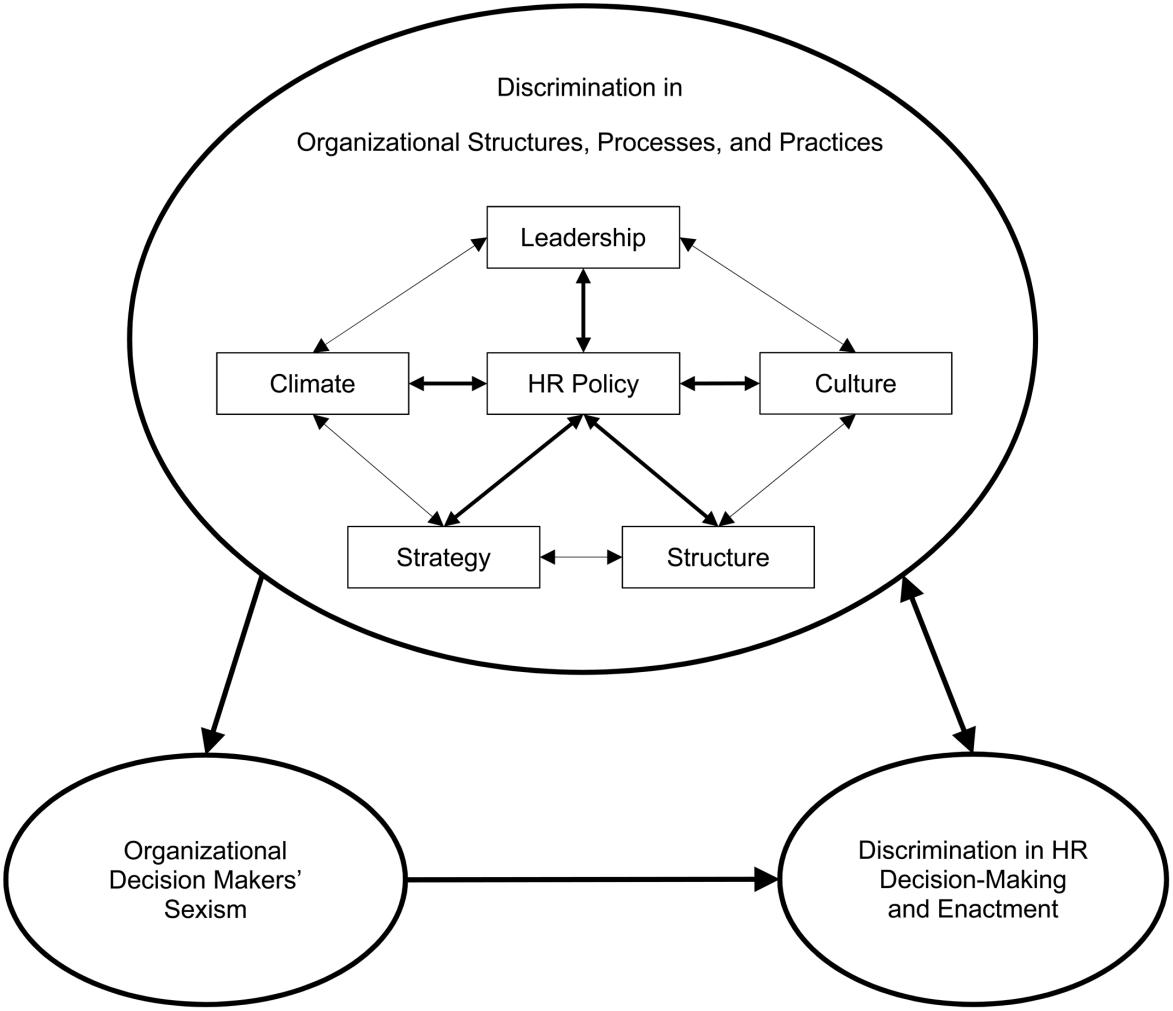
**A model of the root causes of gender discrimination in HR policies, decision-making, and enactment**.

Within this paper, we delineate the nature of discrimination within HR policies, decisions, and their enactment, as well as explore the causes of such discrimination in the workplace. Our model is shown in **Figure 1**. In the Section “Discrimination in HR Related Practices: HR Policy, Decisions, and their Enactment,” we explain the distinction between HR policy, HR-related decision-making, and HR enactment and their relations to each other. Gender inequalities in HR policy are a form of institutional discrimination. We review evidence of institutional discrimination against women within HR policies set out to determine employee selection, performance evaluations, and promotions. In contrast, discrimination in HR-related decisions and their enactment can result from organizational decision makers’ biased responses: it is a form of personal discrimination. Finally, we provide evidence of personal discrimination against women by organizational decision makers in HR-related decision-making and in the enactment of HR policies.

In the Section “The Effect of Organizational Structures, Processes, and Practices on HR Practices,” we focus on the link between institutional discrimination in organizational structures, processes, and practices that can lead to personal discrimination in HR practices (see **Figure 1**). Inspired by the work of Gelfand et al. (2007), we propose that organizational structures, processes, and practices (i.e., leadership, structure, strategy, culture, climate, and HR policy) are interrelated and may contribute to discrimination. Accordingly, gender inequalities in each element can affect the others, creating a self-reinforcing system that can perpetuate institutional discrimination throughout the organization and that can lead to discrimination in HR policies, decision-making, and enactment. We also propose that these relations between gender inequalities in the organizational structures, processes, and practices and discrimination in HR practices can be bidirectional (see **Figure 1**). Thus, we also review how HR practices can contribute to gender inequalities in organizational structures, processes, and practices.

In the Section “The Effect of Hostile and Benevolent Sexism on How Organizational Decision Makers’ Conduct HR Practices,” we delineate the link between organizational decision makers’ levels of sexism and their likelihood of making gender-biased HR-related decisions and/or behaving in a sexist manner when enacting HR policies (e.g., engaging in gender harassment). We focus on two forms of sexist attitudes: hostile and benevolent sexism (Glick and Fiske, 1996). Hostile sexism involves antipathy toward, and negative stereotypes about, agentic women. In contrast, benevolent sexism involves positive but paternalistic views of women as highly communal. Whereas previous research on workplace discrimination has focused on forms of sexism that are hostile in nature, we extend this work by explaining how benevolent sexism, which is more subtle, can also contribute in meaningful yet distinct ways to gender discrimination in HR practices.

In the Section “The Effect of Organizational Structures, Processes, and Practices on Organizational Decision Makers’ Levels of Hostile and Benevolent Sexism,” we describe how institutional discrimination in organizational structures, processes, and practices play a critical role in our model because not only do they affect HR-related decisions and the enactment of HR policies, they also provide a socializing context for organizational decision makers’ levels of hostile and benevolent sexism. In other words, where more institutional discrimination is present, we can expect higher levels of sexism—a third link in our model—which leads to gender bias in HR practices.

In the Section “How to Reduce Gender Discrimination in Organizations,” we discuss how organizations can reduce gender discrimination. We suggest that, to reduce discrimination, organizations should focus on: HR practices, other closely related organizational structures, processes, and practices, and the reduction of organizational decision makers’ level of sexism. Organizations should take such a multifaceted approach because, consistent with our model, gender discrimination is a result of a complex interplay between these factors. Therefore, a focus on only one factor may not be as effective if all the other elements in the model continue to promote gender inequality.

The model we propose for understanding gender inequalities at work is, of course, limited and not intended to be exhaustive. First, we only focus on women’s experience of discrimination. Although men also face discrimination, the focus of this paper is on women because they are more often targets (Branscombe, 1998; Schmitt et al., 2002; McLaughlin et al., 2012) and discrimination is more psychologically damaging for women than for men (Barling et al., 1996; Schmitt et al., 2002). Furthermore, we draw on research from Western, individualistic countries conducted between the mid-1980s to the mid-2010s that might not generalize to other countries or time frames. In addition, this model derives from research that has been conducted primarily in sectors dominated by men. This is because gender discrimination (Mansfield et al., 1991; Welle and Heilman, 2005) and harassment (Mansfield et al., 1991; Berdhal, 2007) against women occur more in environments dominated by men. Now that we have outlined the sections of the paper and our model, we now turn to delineating how gender discrimination in the workplace can be largely attributed to HR practices.

## Discrimination in HR Related Practices: HR Policy, Decisions, and their Enactment

In this section, we explore the nature of gender discrimination in HR practices, which involves HR policies, HR-related decision-making, and their enactment by organizational decision makers. HR is a system of organizational practices aimed at managing employees and ensuring that they are accomplishing organizational goals (Wright et al., 1994). HR functions include: selection, performance evaluation, leadership succession, and training. Depending on the size and history of the organization, HR systems can range from those that are well structured and supported by an entire department, led by HR specialists, to haphazard sets of policies and procedures enacted by managers and supervisors without formal training. HR practices are critically important because they determine the access employees have to valued reward and outcomes within an organization, and can also influence their treatment within an organization (Levitin et al., 1971).

Human resource practices can be broken down into formal HR policy, HR-related decision-making, and the enactment of HR policies and decisions. HR policy codifies practices for personnel functions, performance evaluations, employee relations, and resource planning (Wright et al., 1994). HR-related decision-making occurs when organizational decision makers (i.e., managers, supervisors, or HR personnel) employ HR policy to determine how it will be applied to a particular situation and individual. The enactment of HR involves the personal interactions between organizational decision makers and job candidates or employees when HR policies are applied. Whereas HR policy can reflect institutional discrimination, HR-related decision-making and enactment can reflect personal discrimination by organizational decision makers.

### Institutional Discrimination in HR Policy

Human resource policies that are inherently biased against a group of people, regardless of their job-related knowledge, skills, abilities, and performance can be termed institutional discrimination. Institutional discrimination against women can occur in each type of HR policy from the recruitment and selection of an individual into an organization, through his/her role assignments, training, pay, performance evaluations, promotion, and termination. For instance, if women are under-represented in a particular educational program or a particular job type and those credentials or previous job experience are required to be considered for selection, women are being systematically, albeit perhaps not intentionally, discriminated against. In another example, there is gender discrimination if a test is used in the selection battery for which greater gender differences emerge, than those that emerge for job performance ratings (Hough et al., 2001). Thus, institutional discrimination can be present within various aspects of HR selection policy, and can negatively affect women’s work outcomes.

Institutional discrimination against women also occurs in performance evaluations that are used to determine organizational rewards (e.g., compensation), opportunities (e.g., promotion, role assignments), and punishments (e.g., termination). Gender discrimination can be formalized into HR policy if criteria used by organizational decision makers to evaluate job performance systematically favor men over women. For instance, “face time” is a key performance metric that rewards employees who are at the office more than those who are not. Given that women are still the primary caregivers (Acker, 1990; Fuegen et al., 2004), women use flexible work arrangements more often than men and, consequently, face career penalties because they score lower on face time (Glass, 2004). Thus, biased criteria in performance evaluation policies can contribute to gender discrimination.

Human resource policies surrounding promotions and opportunities for advancement are another area of concern. In organizations with more formal job ladders that are used to dictate and constrain workers’ promotion opportunities, women are less likely to advance (Perry et al., 1994). This occurs because job ladders tend to be divided by gender, and as such, gender job segregation that is seen at entry-level positions will be strengthened as employees move up their specific ladder with no opportunity to cross into other lines of advancement. Thus, women will lack particular job experiences that are not available within their specific job ladders, making them unqualified for advancement (De Pater et al., 2010).

In sum, institutional discrimination can be present within HR policies set out to determine employee selection, performance evaluations, and promotions. These policies can have significant effects on women’s careers. However, HR policy can only be used to guide HR-related decision-making. In reality, it is organizational decision-makers, that is, managers, supervisors, HR personnel who, guided by policy, must evaluate job candidates or employees and decide how policy will be applied to individuals.

### Personal Discrimination in HR-Related Decision-Making

The practice of HR-related decision-making involves social cognition in which others’ competence, potential, and deservingness are assessed by organizational decision makers. Thus, like all forms of social cognition, HR-related decision-making is open to personal biases. HR-related decisions are critically important because they determine women’s pay and opportunities at work (e.g., promotions, training opportunities). Personal discrimination against women by organizational decision makers can occur in each stage of HR-related decision-making regarding recruitment and selection, role assignments, training opportunities, pay, performance evaluation, promotion, and termination.

Studies with varying methodologies show that women face personal discrimination when going through the selection process (e.g., Goldberg, 1968; Rosen and Jerdee, 1974). Meta-analyses reveal that, when being considered for male-typed (i.e., male dominated, believed-to-be-for-men) jobs, female candidates are evaluated more negatively and recommended for employment less often by study participants, compared with matched male candidates (e.g., Hunter et al., 1982; Tosi and Einbender, 1985; Olian et al., 1988; Davison and Burke, 2000). For example, in audit studies, which involve sending ostensibly real applications for job openings while varying the gender of the applicant, female applicants are less likely to be interviewed or called back, compared with male applicants (e.g., McIntyre et al., 1980; Firth, 1982). In a recent study, male and female biology, chemistry, and physics professors rated an undergraduate science student for a laboratory manager position (Moss-Racusin et al., 2012). The male applicant was rated as significantly more competent and hireable, offered a higher starting salary (about $4000), and offered more career mentoring than the female applicant was. In summary, women face a distinct disadvantage when being considered for male-typed jobs.

There is ample evidence that women experience biased performance evaluations on male-typed tasks. A meta-analysis of experimental studies reveals that women in leadership positions receive lower performance evaluations than matched men; this is amplified when women act in a stereotypically masculine, that is, agentic fashion (Eagly et al., 1992). Further, in masculine domains, women are held to a higher standard of performance than men are. For example, in a study of military cadets, men and women gave their peers lower ratings if they were women, despite having objectively equal qualifications to men (Boldry et al., 2001). Finally, women are evaluated more poorly in situations that involve complex problem solving; in these situations, people are skeptical regarding women’s expertise and discredit expert women’s opinions but give expert men the benefit of the doubt (Thomas-Hunt and Phillips, 2004).

Sometimes particular types of women are more likely to be discriminated against in selection and performance evaluation decisions. Specifically, agentic women, that is, those who behave in an assertive, task-oriented fashion, are rated as less likeable and less hireable than comparable agentic male applicants (Heilman and Okimoto, 2007; Rudman and Phelan, 2008; Rudman et al., 2012). In addition, there is evidence of discrimination against pregnant women when they apply for jobs (Hebl et al., 2007; Morgan et al., 2013). Further, women who are mothers are recommended for promotion less than women who are not mothers or men with or without children (Heilman and Okimoto, 2008). Why might people discriminate specifically against agentic women and pregnant women or mothers, who are seemingly very different? The stereotype content model, accounts for how agentic women, who are perceived to be high in competence and low in warmth, will be discriminated against because of feelings of competition; whereas, pregnant women and mothers, who are seen as low in competence, but high in warmth, will be discriminated against because of a perceived lack of deservingness (Fiske et al., 1999, 2002; Cuddy et al., 2004). Taken together, research has uncovered that different forms of bias toward specific subtypes of women have the same overall effect—bias in selection and performance evaluation decisions.

Women are also likely to receive fewer opportunities at work, compared with men, resulting in their under-representation at higher levels of management and leadership within organizations (Martell et al., 1996; Eagly and Carli, 2007). Managers give women fewer challenging roles and fewer training opportunities, compared with men (King et al., 2012; Glick, 2013). For instance, female managers (Lyness and Thompson, 1997) and midlevel workers (De Pater et al., 2010) have less access to high-level responsibilities and challenges that are precursors to promotion. Further, men are more likely to be given key leadership assignments in male-dominated fields *and in female-dominated fields* (e.g., Maume, 1999; De Pater et al., 2010). This is detrimental given that challenging roles, especially developmental ones, help employees gain important skills needed to excel in their careers (Spreitzer et al., 1997).

Furthermore, managers rate women as having less promotion potential than men (Roth et al., 2012). Given the same level of qualifications, managers are less likely to grant promotions to women, compared with men (Lazear and Rosen, 1990). Thus, men have a faster ascent in organizational hierarchies than women (Cox and Harquail, 1991; Stroh et al., 1992; Blau and DeVaro, 2007). Even minimal amounts of gender discrimination in promotion decisions for a particular job or level can have large, cumulative effects given the pyramid structure of most hierarchical organizations (Martell et al., 1996; Baxter and Wright, 2000). Therefore, discrimination by organizational decision makers results in the under-promotion of women.

Finally, women are underpaid, compared with men. In a comprehensive US study using data from 1983 to 2000, after controlling for human capital factors that could affect wages (e.g., education level, work experience), the researchers found that women were paid 22% less than men (U.S. Government Accountability Office, 2003). Further, within any given occupation, men typically have higher wages than women; this “within-occupation” wage gap is especially prominent in more highly paid occupations (U.S. Census Bureau, 2007). In a study of over 2000 managers, women were compensated less than men were, even after controlling for a number of human capital factors (Ostroff and Atwater, 2003). Experimental work suggests that personal biases by organizational decision makers contribute to the gender wage gap. When participants are asked to determine starting salaries for matched candidates that differ by gender, they pay men more (e.g., Steinpreis et al., 1999; Moss-Racusin et al., 2012). Such biases are consequential because starting salaries determine life-time earnings (Gerhart and Rynes, 1991). In experimental studies, when participants evaluate a man vs. a woman who is matched on job performance, they choose to compensate men more (Marini, 1989; Durden and Gaynor, 1998; Lips, 2003). Therefore, discrimination in HR-related decision-making by organizational decision makers can contribute to women being paid less than men are.

Taken together, we have shown that there is discrimination against women in decision-making related to HR. These biases from organizational decision makers can occur in each stage of HR-related decision-making and these biased HR decisions have been shown to negatively affect women’s pay and opportunities at work. In the next section, we review how biased HR practices are enacted, which can involve gender harassment.

### Personal Discrimination in HR Enactment

By HR enactment, we refer to those situations where current or prospective employees go through HR processes or when they receive news of their outcomes from organizational decision makers regarding HR-related issues. Personal gender discrimination can occur when employees are given sexist messages, by organizational decision makers, related to HR enactment. More specifically, this type of personal gender discrimination is termed gender harassment, and consists of a range of verbal and non-verbal behaviors that convey sexist, insulting, or hostile attitudes about women (Fitzgerald et al., 1995a,b). Gender harassment is the most common form of sex-based discrimination (Fitzgerald et al., 1988; Schneider et al., 1997). For example, across the military in the United States, 52% of the 9,725 women surveyed reported that they had experienced gender harassment in the last year (Leskinen et al., 2011, Study 1). In a random sample of attorneys from a large federal judicial circuit, 32% of the 1,425 women attorneys surveyed had experienced gender harassment in the last 5 years (Leskinen et al., 2011, Study 2). When examining women’s experiences of gender harassment, 60% of instances were perpetrated by their supervisor/manager or a person in a leadership role (cf. Crocker and Kalemba, 1999; McDonald et al., 2008). Thus, personal discrimination in the form of gender harassment is a common behavior; however, is it one that organizational decision makers engage in when enacting HR processes and outcomes?

Although it might seem implausible that organizational decision makers would convey sexist sentiments to women when giving them the news of HR-related decisions, there have been high-profile examples from discrimination lawsuits where this has happened. For example, in a class action lawsuit against Walmart, female workers claimed they were receiving fewer promotions than men despite superior qualifications and records of service. In that case, the district manager was accused of confiding to some of the women who were overlooked for promotions that they were passed over because he was not in favor of women being in upper management positions (Wal-Mart Stores, Inc. v. Dukes, 2004/2011). In addition, audit studies, wherein matched men and women apply to real jobs, have revealed that alongside discrimination (McIntyre et al., 1980; Firth, 1982; Moss-Racusin et al., 2012), women experience verbal gender harassment when applying for sex atypical jobs, such as sexist comments as well as skeptical or discouraging responses from hiring staff (Neumark, 1996). Finally, gender harassment toward women when HR policies are enacted can also take the form of offensive comments and denying women promotions due to pregnancy or the chance of pregnancy. For example, in *Moore v. Alabama*, an employee was 8 months pregnant and the woman’s supervisor allegedly looked at her belly and said “I was going to make you head of the office, but look at you now” (Moore v. Alabama State University, 1996, p. 431; Williams, 2003). Thus, organizational decision makers will at times convey sexist sentiments to women when giving them the news of HR-related decisions.

Interestingly, whereas discrimination in HR policy and in HR-related decision-making is extremely difficult to detect (Crosby et al., 1986; Major, 1994), gender harassment in HR enactment provides direct cues to recipients that discrimination is occurring. In other words, although women’s lives are negatively affected in concrete ways by discrimination in HR policy and decisions (e.g., not receiving a job, being underpaid), they may not perceive their negative outcomes as due to gender discrimination. Indeed, there is a multitude of evidence that women and other stigmatized group members are loath to make attributions to discrimination (Crosby, 1984; Vorauer and Kumhyr, 2001; Stangor et al., 2003) and instead are likely to make internal attributions for negative evaluations unless they are certain the evaluator is biased against their group (Ruggiero and Taylor, 1995; Major et al., 2003). However, when organizational decision makers engage in gender harassment during HR enactment women should be more likely to interpret HR policy and HR-related decisions as discriminatory.

Now that we have specified the nature of institutional gender discrimination in HR policy and personal discrimination in HR-related decision-making and in HR enactment, we turn to the issue of understanding the causes of such discrimination: gender discrimination in organizational structures, processes, and practices, and personal biases of organizational decision makers.

## The Effect of Organizational Structures, Processes, and Practices on HR Practices

The first contextual factor within which gender inequalities can be institutionalized is leadership. Leadership is a process wherein an individual (e.g., CEOs, managers) influences others in an effort to reach organizational goals (Chemers, 1997; House and Aditya, 1997). Leaders determine and communicate what the organization’s priorities are to all members of the organization. Leaders are important as they affect the other organizational structures, processes, and practices. Specifically, leaders set culture, set policy, set strategy, and are role models for socialization. We suggest that one important way institutional gender inequality in leadership exists is when women are under-represented, compared with men—particularly when women are well-represented at lower levels within an organization.

An underrepresentation of women in leadership can be perpetuated easily because the gender of organizational leaders affects the degree to which there is gender discrimination, gender supportive policies, and a gender diversity supportive climate within an organization (Ostroff et al., 2012). Organizational members are likely to perceive that the climate for women is positive when women hold key positions in the organization (Konrad et al., 2010). Specifically, the presence of women in key positions acts as a vivid symbol indicating that the organization supports gender diversity. Consistent with this, industries that have fewer female high status managers have a greater gender wage gap (Cohen and Huffman, 2007). Further, women who work with a male supervisor perceive less organizational support, compared with those who work with a female supervisor (Konrad et al., 2010). In addition, women who work in departments that are headed by a man report experiencing more gender discrimination, compared with their counterparts in departments headed by women (Konrad et al., 2010). Some of these effects may be mediated by a similar-to-me bias (Tsui and O’Reilly, 1989), where leaders set up systems that reward and promote individuals like themselves, which can lead to discrimination toward women when leaders are predominantly male (Davison and Burke, 2000; Roth et al., 2012). Thus, gender inequalities in leadership affect women’s experiences in the workplace and their likelihood of facing discrimination.

The second contextual factor to consider is organizational structure. The formal structure of an organization is how an organization arranges itself and it consists of employee hierarchies, departments, etc. (Grant, 2010). An example of institutional discrimination in the formal structure of an organization are job ladders, which are typically segregated by gender (Perry et al., 1994). Such gender-segregated job ladders typically exist within different departments of the organization. Women belonging to gender-segregated networks within organizations (Brass, 1985) have less access to information about jobs, less status, and less upward mobility within the organization (Ragins and Sundstrom, 1989; McDonald et al., 2009). This is likely because in gender-segregated networks, women have less visibility and lack access to individuals with power (Ragins and Sundstrom, 1989). In gender-segregated networks, it is also difficult for women to find female mentors because there is a lack of women in high-ranking positions (Noe, 1988; Linehan and Scullion, 2008). Consequently, the organizational structure can be marked by gender inequalities that reduce women’s chances of reaching top-level positions in an organization.

Gender inequalities can be inherent in the structure of an organization when there are gender segregated departments, job ladders, and networks, which are intimately tied to gender discrimination in HR practices. For instance, if HR policies are designed such that pay is determined based on comparisons between individuals only within a department (e.g., department-wide reporting structure, job descriptions, performance evaluations), then this can lead to a devaluation of departments dominated by women. The overrepresentation of women in certain jobs leads to the lower status of those jobs; consequently, the pay brackets for these jobs decrease over time as the number of women in these jobs increase (e.g., Huffman and Velasco, 1997; Reilly and Wirjanto, 1999). Similarly, networks led by women are also devalued for pay. For example, in a study of over 2,000 managers, after controlling for performance, the type of job, and the functional area (e.g., marketing, sales, accounting), those who worked with female mangers had lower wages than those who worked with male managers (Ostroff and Atwater, 2003). Thus, gender inequalities in an organization’s structure in terms of gender segregation have reciprocal effects with gender discrimination in HR policy and decision-making.

Another contextual factor in our model is organizational strategy and how institutional discrimination within strategy is related to discrimination in HR practices. Strategy is a plan, method, or process by which an organization attempts to achieve its objectives, such as being profitable, maintaining and expanding its consumer base, marketing strategy, etc. (Grant, 2010). Strategy can influence the level of inequality within an organization (Morrison and Von Glinow, 1990; Hunter et al., 2001). For example, Hooters, a restaurant chain, has a marketing strategy to sexually attract heterosexual males, which has led to discrimination in HR policy, decisions, and enactment because only young, good-looking women are considered qualified (Schneyer, 1998). When faced with appearance-based discrimination lawsuits regarding their hiring policies, Hooters has responded by claiming that such appearance requirements are bona fide job qualifications given their marketing strategy (for reviews, see Schneyer, 1998; Adamitis, 2000). Hooters is not alone, as many other establishments attempt to attract male cliental by requiring their female servers to meet a dress code involving a high level of grooming (make-up, hair), a high heels requirement, and a revealing uniform (McGinley, 2007). Thus, sexist HR policies and practices in which differential standards are applied to male and female employees can stem from a specific organizational strategy (Westall, 2015).

We now consider institutional gender bias within organizational culture and how it relates to discrimination in HR policies. Organizational culture refers to collectively held beliefs, assumptions, and values held by organizational members (Trice and Beyer, 1993; Schein, 2010). Cultures arise from the values of the founders of the organization and assumptions about the right way of doing things, which are learned from dealing with challenges over time (Ostroff et al., 2012). The founders and leaders of an organization are the most influential in forming, maintaining, and changing culture over time (e.g., Trice and Beyer, 1993; Jung et al., 2008; Hartnell and Walumbwa, 2011). Organizational culture can contribute to gender inequalities because culture constrains people’s ideas of what is possible: their strategies of action (Swidler, 1986). In other words, when people encounter a problem in their workplace, the organizational culture—who we are, how we act, what is right—will provide only a certain realm of behavioral responses. For instance, in organizational cultures marked by greater gender inequality, women may have lower hopes and expectations for promotion, and when they are discriminated against, may be less likely to imagine that they can appeal their outcomes (Kanter, 1977; Cassirer and Reskin, 2000). Furthermore, in organizational cultures marked by gender inequality, organizational decision makers should hold stronger descriptive and proscriptive gender stereotypes: they should more strongly believe that women have less ability to lead, less career commitment, and less emotional stability, compared with men (Eagly et al., 1992; Heilman, 2001). We expand upon this point later.

Other aspects of organizational culture that are less obviously related to gender can also lead to discrimination in HR practices. For instance, an organizational culture that emphasizes concerns with meritocracy, can lead organizational members to oppose HR efforts to increase gender equality. This is because when people believe that outcomes ought to go only to those who are most deserving, it is easy for them to fall into the trap of believing that outcomes currently do go to those who are most deserving (Son Hing et al., 2011). Therefore, people will believe that men deserve their elevated status and women deserve their subordinated status at work (Castilla and Benard, 2010). Furthermore, the more people care about merit-based outcomes, the more they oppose affirmative action and diversity initiatives for women (Bobocel et al., 1998; Son Hing et al., 2011), particularly when they do not recognize that discrimination occurs against women in the absence of such policies (Son Hing et al., 2002). Thus, a particular organizational culture can influence the level of discrimination against women in HR and prevent the adoption of HR policies that would mitigate gender discrimination.

Finally, gender inequalities can be seen in organizational climates. An organizational climate consists of organizational members’ shared perceptions of the formal and informal organizational practices, procedures, and routines (Schneider et al., 2011) that arise from direct experiences of the organization’s culture (Ostroff et al., 2012). Organizational climates tend to be conceptualized and studied as “climates for” an organizational strategy (Schneider, 1975; Ostroff et al., 2012). Gender inequalities are most clearly reflected in two forms of climate: climates for diversity and climates for sexual harassment.

A positive climate for diversity exists when organizational members perceive that diverse groups are included, empowered, and treated fairly. When employees perceive a less supportive diversity climate, they perceive greater workplace discrimination (Cox, 1994; Ragins and Cornwall, 2001; Triana and García, 2009), and experience lower organizational commitment and job satisfaction (Hicks-Clarke and Iles, 2000), and higher turnover intentions (Triana et al., 2010). Thus, in organizations with a less supportive diversity climate, women are more likely to leave the organization, which contributes to the underrepresentation of women in already male-dominated arenas (Miner-Rubino and Cortina, 2004).

A climate for sexual harassment involves perceptions that the organization is permissive of sexual harassment. In organizational climates that are permissive of harassment, victims are reluctant to come forward because they believe that their complaints will not be taken seriously (Hulin et al., 1996) and will result in negative personal consequences (e.g., Offermann and Malamut, 2002). Furthermore, men with a proclivity for harassment are more likely to act out these behaviors when permissive factors are present (Pryor et al., 1993). Therefore, a permissive climate for sexual harassment can result in more harassing behaviors, which can lead women to disengage from their work and ultimately leave the organization (Kath et al., 2009).

Organizational climates for diversity and for sexual harassment are inextricably linked to HR practices. For instance, a factor that leads to perceptions of diversity climates is whether the HR department has diversity training (seminars, workshops) and how much time and money is devoted to diversity efforts (Triana and García, 2009). Similarly, a climate for sexual harassment depends on organizational members’ perceptions of how strict the workplace’s sexual harassment policy is, and how likely offenders are to be punished (Fitzgerald et al., 1995b; Hulin et al., 1996). Thus, HR policies, decision-making, and their enactment strongly affect gender inequalities in organizational climates and gender inequalities throughout an organization.

In summary, gender inequalities can exist within organizational structures, processes, and practices. However, organizational leadership, structure, strategy, culture, and climate do not inherently need to be sexist. It could be possible for these organizational structures, processes, and practices to promote gender equality. We return to this issue in the conclusion section.

## The Effect of Hostile and Benevolent Sexism on How Organizational Decision Makers’ Conduct HR Practices

In this section, we explore how personal biases can affect personal discrimination in HR-related decisions and their enactment. Others have focused on how negative or hostile attitudes toward women predict discrimination in the workplace. However, we extend this analysis by drawing on ambivalent sexism theory, which involves hostile sexism (i.e., antagonistic attitudes toward women) and benevolent sexism (i.e., paternalistic attitudes toward women; see also Glick, 2013), both of which lead to discrimination against women.

Stereotyping processes are one possible explanation of how discrimination against women in male-typed jobs occurs and how women are relegated to the “pink ghetto” (Heilman, 1983; Eagly and Karau, 2002; Rudman et al., 2012). Gender stereotypes, that is, expectations of what women and men are like, and what they should be like, are one of the most powerful schemas activated when people encounter others (Fiske et al., 1991; Stangor et al., 1992). According to status characteristics theory, people’s group memberships convey important information about their status and their competence on specific tasks (Berger et al., 1974; Berger et al., 1998; Correll and Ridgeway, 2003). Organizational decision makers will, for many jobs, have different expectations for men’s and women’s competence and job performance. Expectations of stereotyped-group members’ success can affect gender discrimination that occurs in HR-related decisions and enactment (Roberson et al., 2007). For example, men are preferred over women for masculine jobs and women are preferred over men for feminine jobs (Davison and Burke, 2000). Thus, the more that a workplace role is inconsistent with the attributes ascribed to women, the more a particular woman might be seen as lacking “fit” with that role, resulting in decreased performance expectations (Heilman, 1983; Eagly and Karau, 2002).

Furthermore, because women are associated with lower status, and men with higher status, women experience backlash for pursuing high status roles (e.g., leadership) in the workplace (Rudman et al., 2012). In other words, agentic women who act competitively and confidently in a leadership role, are rated as more socially deficient, less likeable and less hireable, compared with men who act the same way (Rudman, 1998; Rudman et al., 2012). Interestingly though, if women pursue roles in the workplace that are congruent with traditional gender expectations, they will elicit positive reactions (Eagly and Karau, 2002).

Thus, cultural, widely known, gender stereotypes can affect HR-related decisions. However, such an account does not take into consideration individual differences among organizational decision makers (e.g., managers, supervisors, or HR personnel) who may vary in the extent to which they endorse sexist attitudes or stereotypes. Individual differences in various forms of sexism (e.g., modern sexism, neosexism) have been demonstrated to lead to personal discrimination in the workplace (Hagen and Kahn, 1975; Beaton et al., 1996; Hitlan et al., 2009). Ambivalent sexism theory builds on earlier theories of sexism by including attitudes toward women that, while sexist, are often experienced as positive in valence by perceivers and targets (Glick and Fiske, 1996). Therefore, we draw on ambivalent sexism theory, which conceptualizes sexism as a multidimensional construct that encompasses both hostile and benevolent attitudes toward women (Glick and Fiske, 1996, 2001).

Hostile sexism involves antipathy and negative stereotypes about women, such as beliefs that women are incompetent, overly emotional, and sexually manipulative. Hostile sexism also involves beliefs that men should be more powerful than women and fears that women will try to take power from men (Glick and Fiske, 1996; Cikara et al., 2008). In contrast, benevolent sexism involves overall positive views of women, as long as they occupy traditionally feminine roles. Individuals with benevolently sexist beliefs characterize women as weak and needing protection, support, and adoration. Importantly, hostile and benevolent sexism tend to go hand-in-hand (with a typical correlation of 0.40; Glick et al., 2000). This is because ambivalent sexists, people who are high in benevolent and hostile sexism, believe that women should occupy restricted domestic roles and that women are weaker than men are (Glick and Fiske, 1996). Ambivalent sexists reconcile their potentially contradictory attitudes about women by acting hostile toward women whom they believe are trying to steal men’s power (e.g., feminists, professionals who show competence) and by acting benevolently toward traditional women (e.g., homemakers) who reinforce conventional gender relations and who serve men (Glick et al., 1997). An individual difference approach allows us to build on the earlier models (Heilman, 1983; Eagly and Karau, 2002; Rudman et al., 2012), by specifying who is more likely to discriminate against women and why.

Organizational decision makers who are higher (vs. lower) in hostile sexism should discriminate more against women in HR-related decisions (Glick et al., 1997; Masser and Abrams, 2004). For instance, people high in hostile sexism have been found to evaluate candidates, who are believed to be women, more negatively and give lower employment recommendations for a management position, compared with matched candidates believed to be men (Salvaggio et al., 2009)^1^. In another study, among participants who evaluated a female candidate for a managerial position, those higher in hostile sexism were less likely to recommend her for hire, compared with those lower in hostile sexism (Masser and Abrams, 2004). Interestingly, among those evaluating a matched man for the same position, those higher (vs. lower) in hostile sexism were more likely to recommend him for hire (Masser and Abrams, 2004). According to ambivalent sexism theorists (Glick et al., 1997), because people high in hostile sexism see women as a threat to men’s status, they act as gatekeepers denying women access to more prestigious or masculine jobs.

Furthermore, when enacting HR policies and decisions, organizational decision makers who are higher (vs. lower) in hostile sexism should discriminate more against women in the form of gender harassment. Gender harassment can involve hostile terms of address, negative comments regarding women in management, sexist jokes, and sexist behavior (Fitzgerald et al., 1995a,b). It has been found that people higher (vs. lower) in hostile sexism have more lenient attitudes toward the sexual harassment of women, which involves gender harassment, in the workplace (Begany and Milburn, 2002; Russell and Trigg, 2004). Furthermore, men who more strongly believe that women are men’s adversaries tell more sexist jokes to a woman (Mitchell et al., 2004). Women also report experiencing more incivility (i.e., low level, rude behavior) in the workplace than men (Björkqvist et al., 1994; Cortina et al., 2001, 2002), which could be due to hostile attitudes toward women. In summary, the evidence is consistent with the idea that organizational decision makers’ hostile sexism should predict their gender harassing behavior during HR enactment; however, more research is needed for such a conclusion.

In addition, organizational decision makers who are higher (vs. lower) in benevolent sexism should discriminate more against women when making HR-related decisions. It has been found that people higher (vs. lower) in benevolent sexism are more likely to automatically associate men with high-authority and women with low-authority roles and to implicitly stereotype men as agentic and women as communal (Rudman and Kilianski, 2000). Thus, organizational decision makers who are higher (vs. lower) in benevolent sexism should more strongly believe that women are unfit for organizational roles that are demanding, challenging, and requiring agentic behavior. Indeed, in studies of male MBA students those higher (vs. lower) in benevolent sexism assigned a fictional woman less challenging tasks than a matched man (King et al., 2012). The researchers reasoned that this occurred because men are attempting to “protect” women from the struggles of challenging work. Although there has been little research conducted that has looked at benevolent sexism and gender discrimination in HR-related decisions, the findings are consistent with our model.

Finally, organizational decision makers who are higher (vs. lower) in benevolent sexism should engage in a complex form of gender discrimination when enacting HR policy and decisions that involves mixed messages: women are more likely to receive messages of positive verbal feedback (e.g., “stellar work,” “excellent work”) but lower numeric ratings on performance appraisals, compared with men (Biernat et al., 2012). It is proposed that this pattern of giving women positive messages about their performance while rating them poorly reflects benevolent sexists’ desire to protect women from harsh criticism. However, given that performance appraisals are used for promotion decisions and that constructive feedback is needed for learning, managers’ unwillingness to give women negative verbal criticisms can lead to skill plateau and career stagnation.

Furthermore, exposure to benevolent sexism can harm women’s motivation, goals and performance. Adolescent girls whose mothers are high in benevolent (but not hostile) sexism display lower academic goals and academic performance (Montañés et al., 2012). Of greater relevance to the workplace, when role-playing a job candidate, women who interacted with a hiring manager scripted to make benevolently sexist statements became preoccupied with thoughts about their incompetence, and consequently performed worse in the interview, compared with those in a control condition (Dardenne et al., 2007). These findings suggest that benevolent sexism during the enactment of HR practices can harm women’s work-related motivation and goals, as well as their performance, which can result in a self-fulfilling prophecy (Word et al., 1974). In other words, the low expectations benevolent sexists have of women can be confirmed by women as they are undermined by paternalistic messages.

Ambivalent sexism can operate to harm women’s access to jobs, opportunities for development, ratings of performance, and lead to stigmatization. However, hostile and benevolent sexism operate in different ways. Hostile sexism has direct negative consequences for women’s access to high status, male-typed jobs (Masser and Abrams, 2004; Salvaggio et al., 2009), and it is related to higher rates of sexual harassment (Fitzgerald et al., 1995b; Mitchell et al., 2004; Russell and Trigg, 2004), which negatively affect women’s health, well-being, and workplace withdrawal behaviors (Willness et al., 2007). In contrast, benevolent sexism has indirect negative consequences for women’s careers, for instance, in preventing access to challenging tasks (King et al., 2012) and critical developmental feedback (Vescio et al., 2005). Interestingly, exposure to benevolent sexism results in worsened motivation and cognitive performance, compared with exposure to hostile sexism (Dardenne et al., 2007; Montañés et al., 2012). This is because women more easily recognize hostile sexism as a form of discrimination and inequality, compared with benevolent sexism, which can be more subtle in nature (Dardenne et al., 2007). Thus, women can externalize hostile sexism and mobilize against it, but the subtle nature of benevolent sexism prevents these processes (Kay et al., 2005; Becker and Wright, 2011). Therefore, hostile and benevolent sexism lead to different but harmful forms of HR discrimination. Future research should more closely examine their potentially different consequences.

Thus far, we have articulated how gender inequalities in organizational structures, processes, and practices can affect discrimination in HR policy and in HR-related decision-making and enactment. Furthermore, we have argued that organizational decision makers’ levels of hostile and benevolent sexism are critical factors leading to personal discrimination in HR-related decision-making and enactment, albeit in different forms. We now turn to an integration of these two phenomena.

## The Effect of Organizational Structures, Processes, and Practices on Organizational Decision Makers’ Levels of Hostile and Benevolent Sexism

Organizational decision makers’ beliefs about men and women should be affected by the work environments in which they are embedded. Thus, when there are more gender inequalities within organizational structures, processes, and practices, organizational decision makers should have higher levels of hostile sexism and benevolent sexism. Two inter-related processes can account for this proposition: the establishment of who becomes and remains an organizational member, and the socialization of organizational members.

First, as organizations develop over time, forces work to attract, select, and retain an increasingly homogenous set of employees in terms of their hostile and benevolent sexism (Schneider, 1983, 1987). In support of this perspective, an individual’s values tend to be congruent with the values in his or her work environment (e.g., Holland, 1996; Kristof-Brown et al., 2005). People are attracted to and choose to work for organizations that have characteristics similar to their own, and organizations select individuals who are likely to fit with the organization. Thus, more sexist individuals are more likely to be attracted to organizations with greater gender inequality in leadership, structure, strategy, culture, climate, and HR policy; and they will be seen as a better fit during recruitment and selection. Finally, individuals who do not fit with the organization tend to leave voluntarily through the process of attrition. Thus, less (vs. more) sexist individuals would be more likely to leave a workplace with marked gender inequalities in organizational structures, processes, and practices. The opposite should be true for organizations with high gender equality. Through attraction, selection, and attrition processes it is likely that organizational members will become more sexist in a highly gender unequal organization and less sexist in a highly gender equal organization.

Second, socialization processes can change organizational members’ personal attributes, goals, and values to match those of the organization (Ostroff and Rothausen, 1997). Organizational members’ receive both formal and informal messages about gender inequality—or equality—within an organization through their orientation and training, reading of organizational policy, perceptions of who rises in the ranks, how women (vs. men) are treated within the organization, as well as their perception of climates for diversity and sexual harassment. Socialization of organizational members over time has been shown to result in organizational members’ values and personalities changing to better match the values of the organization (Kohn and Schooler, 1982; Cable and Parsons, 2001).

These socialization processes can operate to change organizational members’ levels of sexism. It is likely that within more sexist workplaces, people’s levels of hostile and benevolent sexism increase because their normative beliefs shift due to exposure to institutional discrimination against women, others’ sexist attitudes and behavior, and gender bias in culture and climate (Schwartz and DeKeseredy, 2000; Ford et al., 2008; Banyard et al., 2009). These processes can also lead organizational decision makers to adopt less sexist attitudes in a workplace context marked by greater gender equality. Thus, organizational members’ levels of hostile and benevolent sexism can be shaped by the degree of gender inequalities in organizational structures, processes, and practices and by the sexism levels of their work colleagues.

In addition, organizational decision makers can be socialized to act in discriminatory ways without personally becoming more sexist. If organizational decision makers witness others acting in a discriminatory manner with positive consequences, or acting in an egalitarian way with negative consequences, they can learn to become more discriminatory in their HR practices through observational learning (Bandura, 1977, 1986). So, organizational decision makers could engage in personal discrimination without being sexist if they perceive that the fair treatment of women in HR would encounter resistance given the broader organizational structures, processes, and practices promoting gender inequality. Yet over time, given cognitive dissonance (Festinger, 1962), it is likely that discriminatory behavior could induce attitude change among organizational decision makers to become more sexist.

Thus far we have argued that gender inequalities in organizational structures, processes, and practices, organizational decision makers’ sexist attitudes, and gender discrimination in HR practices can have reciprocal, reinforcing relationships. Thus, it may appear that we have created a model that is closed and determinate in nature; however, this would be a misinterpretation. In the following section, we outline how organizations marked by gender inequalities can reduce discrimination against women.

## How to Reduce Gender Discrimination in Organizations

The model we present for understanding gender discrimination in HR practices is complex. We believe that such complexity is necessary to accurately reflect the realities of organizational life. The model demonstrates that many sources of gender inequality are inter-related and have reciprocal effects. By implication, there are no simple or direct solutions to reduce gender discrimination in organizations. Rather, this complex problem requires multiple solutions. In fact, as discussed by Gelfand et al. (2007), if an organization attempts to correct discrimination in only one aspect of organizational structure, process, or practice, and not others, such change attempts will be ineffective due to mixed messages. Therefore, we outline below how organizations can reduce gender discrimination by focusing on (a) HR policies (i.e., diversity initiatives and family friendly policies) and closely related organizational structures, processes, and practices; (b) HR-related decision-making and enactment; as well as, (c) the organizational decision makers who engage in such actions.

### Reducing Gender Discrimination in HR Policy and Associated Organizational Structures, Processes, and Practices

Organizations can take steps to mitigate discrimination in HR policies. As a first example, let us consider how an organization can develop, within its HR systems, diversity initiatives aimed at changing the composition of the workforce that includes policies to recruit, retain, and develop employees from underrepresented groups (Jayne and Dipboye, 2004). Diversity initiatives can operate like affirmative action programs in that organizations track and monitor (a) the number of qualified candidates from different groups (e.g., women vs. men) in a pool, and (b) the number of candidates from each group hired or promoted. When the proportion of candidates from a group successfully selected varies significantly from their proportion in the qualified pool then action, such as targeted recruitment efforts, needs to be taken.

Importantly, such efforts to increase diversity can be strengthened by other HR policies that reward managers, who select more diverse personnel, with bonuses (Jayne and Dipboye, 2004). Organizations that incorporate diversity-based criteria into their performance and promotion policies and offer meaningful incentives to managers to identify and develop successful female candidates for promotion are more likely to succeed in retaining and promoting diverse talent (Murphy and Cleveland, 1995; Cleveland et al., 2000). However, focusing on short-term narrowly defined criteria, such as increasing the number of women hired, without also focusing on candidates’ merit and providing an adequate climate or support for women are unlikely to bring about any long-term change in diversity, and can have detrimental consequences for its intended beneficiaries (Heilman et al., 1992, 1997). Rather, to be successful, HR policies for diversity need to be supported by the other organizational structures, processes, and practices, such as strategy, leadership, and climate.

For instance, diversity initiatives should be linked to strategies to create a business case for diversity (Jayne and Dipboye, 2004). An organization with a strategy to market to more diverse populations can justify that a more diverse workforce can better serve potential clientele (Jayne and Dipboye, 2004). Alternatively, an organization that is attempting to innovate and grow might justify a corporate strategy to increase diversity on the grounds that diverse groups have multiple perspectives on a problem with the potential to generate more novel, creative solutions (van Knippenberg et al., 2004). Furthermore, organizational leaders must convey strong support for the HR policies for them to be successful (Rynes and Rosen, 1995). Given the same HR policy within an organization, leaders’ personal attitudes toward the policy affects the discrimination levels found within their unit (Pryor, 1995; Pryor et al., 1995). Finally, diversity programs are more likely to succeed in multicultural organizations with strong climates for diversity (Elsass and Graves, 1997; Jayne and Dipboye, 2004). An organization’s climate for diversity consists of employees’ shared perceptions that the organization’s structures, processes, and practices are committed to maintaining diversity and eliminating discrimination (Nishii and Raver, 2003; Gelfand et al., 2007). In organizations where employees perceive a strong climate for diversity, diversity programs result in greater employee attraction and retention among women and minorities, at all levels of the organization (Cox and Blake, 1991; Martins and Parsons, 2007).

As a second example of how HR policies can mitigate gender inequalities, we discuss HR policies to lessen employees’ experience of work-family conflict. Work-family conflict is a type of role conflict that workers experience when the demands (e.g., emotional, cognitive, time) of their work role interfere with the demands of their family role or vice versa (Greenhaus and Beutell, 1985). Work-family conflict has the negative consequences of increasing employee stress, illness-related absence, and desire to turnover (Grandey and Cropanzano, 1999). Importantly, women are more adversely affected by work-family conflict than men (Martins et al., 2002). Work-family conflict can be exacerbated by HR policies that evaluate employees based on face time (i.e., number of hours present at the office), as a proxy for organizational commitment (Perlow, 1995; Elsbach et al., 2010).

Formal family friendly HR policies can be adopted to relieve work-family conflict directly, which differentially assists women in the workplace. For instance, to reduce work-family conflict, organizations can implement HR policies such as flexible work arrangements, which involve flexible schedules, telecommuting, compressed work weeks, job-shares, and part-time work (Galinsky et al., 2008). In conjunction with other family friendly policies, such as the provision of childcare, elderly care, and paid maternity leave, organizations can work to reduce stress and improve the retention of working mothers (Burke, 2002).

Unfortunately, it has been found that the enactment of flexible work policies can still lead to discrimination. Organizational decision makers’ sexism can lead them to grant more flexible work arrangements to white men than to women and other minorities because white men are seen as more valuable (Kelly and Kalev, 2006). To circumvent this, organizations need to formalize HR policies relating to flexible work arrangements (Kelly and Kalev, 2006). For instance, formal, written policies should articulate who can adopt flexible work arrangements (e.g., employees in specific divisions or with specific job roles) and what such arrangements look like (e.g., core work from 10 am to 3 pm with flexible work hours from 7 to 10 am or from 3 to 6 pm). When the details of such policies are formally laid out, organizational decision makers have less latitude and therefore less opportunity for discrimination in granting access to these arrangements.

To be successful, family friendly HR policies should be tied to other organizational structures, processes, and practices such as organizational strategy, leadership, culture, and climate. A business case for flexible work arrangements can be made because they attract and retain top-talent, which includes women (Baltes et al., 1999). Furthermore, organizational leaders must convey strong support for family friendly programs (Jayne and Dipboye, 2004). Leaders can help bolster the acceptance of family friendly policies through successive interactions, communications, visibility, and role modeling with employees. For instance, a leader who sends emails at 2 o’clock in the morning is setting a different expectation of constant availability than a leader who never sends emails after 7:00 pm. Family friendly HR policies must also be supported by simultaneously changing the underlying organizational culture that promotes face time. Although it is difficult to change the culture of an organization, the leaders’ of the organization play an influential role in instilling such change because the behaviors of leaders are antecedents and triggers of organizational culture (Kozlowski and Doherty, 1989; Ostroff et al., 2012). In summary, HR policies must be supported by other organizational structures, processes, and practices in order for these policies to be effective.

Adopting HR diversity initiative policies and family friendly policies can reduce gender discrimination and reshape the other organizational structures, processes, and practices and increase gender equality in them. Specifically, such policies, if successful, should increase the number of women in all departments and at all levels of an organization. Further, having more women in leadership positions signals to organizational members that the organization takes diversity seriously, affecting the diversity climate of the organization, and ultimately its culture (Konrad et al., 2010). Thus, particular HR policies can reduce gender inequalities in all of the other organizational structures, processes, and practices.

### Reducing Gender Discrimination in HR-Related Decision-Making and Enactment

A wealth of research demonstrates that an effective means of reducing personal bias by organizational decision makers in HR practices is to develop HR policies that standardize and objectify performance data (e.g., Konrad and Linnehan, 1995; Reskin and McBrier, 2000). To reduce discrimination in personnel decisions (i.e., employee hiring and promotion decisions) a job analysis should be performed to determine the appropriate knowledge skills and abilities needed for specific positions (Fine and Cronshaw, 1999). This ensures that expectations about characteristics of the ideal employee for that position are based on accurate knowledge of the job and not gender stereotypes about the job (Welle and Heilman, 2005). To reduce discrimination in performance evaluations, HR policies should necessitate the use of reliable measures based on explicit objective performance expectations and apply these practices consistently across all worker evaluations (Bernardin et al., 1998; Ittner et al., 2003). Employees’ performance should be evaluated using behaviorally anchored rating scales (Smith and Kendall, 1963) that allow supervisors to rate subordinates on examples of actual work behaviors. These evaluations should be done regularly, given that delays require retrieving memories of work performance and this process can be biased by gender stereotypes (Sanchez and De La Torre, 1996). Finally, if greater gender differences are found on selection tests than on performance evaluations, then the use of such biased selection tests needs to be revisited (Chung-Yan and Cronshaw, 2002). In summary, developing HR policies that standardize and objectify the process of employee/candidate evaluations can reduce personal bias in HR practices.

Importantly, the level of personal discrimination enacted by organizational decision makers can be reduced by formalizing HR policies, and by controlling the situations under which HR-related decisions are made. We have articulated how HR-related decisions involve social cognition and are therefore susceptible to biases introduced by the use of gender stereotypes. This can occur unwittingly by those who perceive themselves to be unprejudiced but who are affected by stereotypes or negative automatic associations nonetheless (Chugh, 2004; Son Hing et al., 2008). For instance, when HR policies do not rely on objective criteria, and the context for evaluation is ambiguous, organizational decision makers will draw on gender (and other) stereotypes to fill in the blanks when evaluating candidates (Heilman, 1995, 2001). Importantly, the context can be constructed in such a way as to reduce these biases. For instance, organizational decision makers will make less biased judgments of others if they have more time available to evaluate others, are less cognitively busy (Martell, 1991), have higher quality of information available about candidates, and are accountable for justifying their ratings and decisions (Kulik and Bainbridge, 2005; Roberson et al., 2007). Thus, if they have the time, motivation, and opportunity to make well-informed, more accurate judgments, then discrimination in performance ratings can be reduced.

### Reducing Organizational Decision Makers’ Sexism

Another means to reduce gender discrimination in HR-related decision-making and enactment is to focus directly on reducing the hostile and benevolent sexist beliefs of organizational decision makers. Interventions aimed at reducing these beliefs typically involve diversity training, such as a seminar, course, or workshop. Such training involves one or more sessions that involve interactive discussions, lectures, and practical assignments. During the training men and women are taught about sexism and how gender roles in society are socially constructed. Investigations have shown these workshop-based interventions are effective at reducing levels of hostile sexism but have inconsistent effects on benevolent sexism (Case, 2007; de Lemus et al., 2014). The subtle, and in some ways positive nature of benevolent sexism makes it difficult to confront and reduce using such interventions. However, levels of benevolent sexism are reduced when individuals are explicitly informed about the harmful implications of benevolent sexism (Becker and Swim, 2012). Unfortunately, these interventions have not been tested in organizational settings. So their efficacy in the field is unknown.

## Conclusion

Gender inequality in organizations is a complex phenomenon that can be seen in HR practices (i.e., policies, decision-making, and their enactment) that affects the hiring, training, pay, and promotion of women. We propose that gender discrimination in HR-related decision-making and the enactment of HR practices stems from gender inequalities in broader organizational structures, processes, and practices, including HR policy but also leadership, structure, strategy, culture, and organizational climate. Moreover, reciprocal effects should occur, such that discriminatory HR practices can perpetuate gender inequalities in organizational leadership, structure, strategy, culture, and climate. Organizational decision makers also play an important role in gender discrimination. We propose that personal discrimination in HR-related decisions and enactment arises from organizational decision makers’ levels of hostile and benevolent sexism. While hostile sexism can lead to discrimination against women because of a desire to keep them from positions of power, benevolent sexism can lead to discrimination against women because of a desire to protect them. Finally, we propose that gender inequalities in organizational structures, processes, and practices affect organizational decision makers’ sexism through attraction, selection, socialization, and attrition processes. Thus, a focus on organizational structure, processes, and practices is critical.

The model we have developed extends previous work by Gelfand et al. (2007) in a number of substantive ways. Gelfand et al. (2007) proposed that aspects of the organization, that is, structure, organizational culture, leadership, strategy, HR systems, and organizational climates, are all interrelated and may contribute to or attenuate discrimination (e.g., racism, sexism, ableism, homophobia). First, we differ from their work by emphasizing that workplace discrimination is most directly attributable to HR practices. Consequently, we emphasize how inequalities in other organizational structures, processes, and practices affect institutional discrimination in HR policy. Second, our model differs from that of Gelfand et al. (2007) in that we focus on the role of organizational decision makers in the enactment of HR policy. The attitudes of these decision makers toward specific groups of employees are critical. However, the nature of prejudice differs depending on the target group (Son Hing and Zanna, 2010). Therefore, we focus on one form of bias—sexism—in the workplace. Doing so, allows us to draw on more nuanced theories of prejudice, namely ambivalent sexism theory (Glick and Fiske, 1996). Thus, third, our model differs from the work of Gelfand et al. (2007) by considering how dual beliefs about women (i.e., hostile and benevolent beliefs) can contribute to different forms of gender discrimination in HR practices. Fourth, we differ from Gelfand et al. (2007) by reviewing how organizational decision makers’ level of sexism within an organization is affected by organizational structures, processes, and practices via selection-attraction-attrition processes and through socialization processes.

However, the model we have developed is not meant to be exhaustive. There are multiple issues that we have not addressed but should be considered: what external factors feed into our model? What other links within the model might arise? What are the limits to its generalizability? What consequences derive from our model? How can change occur given a model that is largely recursive in nature? We focus on these issues throughout our conclusion.

In this paper, we have illustrated what we consider to be the dominant links in our model; however, additional links are possible. First, we do not lay out the factors that feed into our model, such as government regulations, the economy, their competitors, and societal culture. In future work, one could analyze the broader context that organizations operate in, which influences its structures, processes, and practices, as well as its members. For instance, in societies marked by greater gender inequalities, the levels of hostile and benevolent sexism of organizational decision makers will be higher (Glick et al., 2000). Second, there is no link demonstrating how organizational decision makers who are more sexist have the capacity, even if they sit lower in the organizational hierarchy, to influence the amount of gender inequality in organizational structures, processes, and practices. It is possible for low-level managers or HR personnel who express more sexist sentiments to—through their own behavior—affect others’ perceptions of the tolerance for discrimination in the workplace (Ford et al., 2001) and others’ perceptions of the competence and hireability of female job candidates (Good and Rudman, 2010). Thus, organizational decision makers’ levels of hostile and benevolent sexism can affect organizational climates, and potentially other organizational structures, processes, and practices. Third, it is possible that organizational structures, processes, and practices could moderate the link between organizational decision makers’ sexist attitudes and their discriminatory behavior in HR practices. The ability of people to act in line with their attitudes depends on the strength of the constraints in the social situation and the broader context (Lewin, 1935, 1951). Thus, if organizational structures, processes, and practices clearly communicate the importance of gender equality then the discriminatory behavior of sexist organizational decision makers should be constrained. Accordingly, organizations should take steps to mitigate institutional discrimination by focusing on organizational structures, processes, and practices rather than focusing solely on reducing sexism in individual employees.

Our model does not consider how women’s occupational status is affected by their preferences for gender-role-consistent careers and their childcare and family responsibilities, which perhaps should not be underestimated (e.g., Manne, 2001; Hakim, 2006; Ceci et al., 2009). In other words, lifestyle preferences could contribute to gender differences in the workplace. However, it is important to consider how women’s agency in choosing occupations and managing work-life demands is constrained. Gender imbalances (e.g., in pay) in the workplace (e.g., Moss-Racusin et al., 2012; Sheltzer and Smith, 2014) and gender imbalances in the home (e.g., in domestic labor, childcare; Bianchi, 2000; Bianchi et al., 2000) shape the decisions that couples (when they consist of a woman and a man) make about how to manage dual careers. For instance, research has uncovered that women with professional degrees leave the labor force at roughly three times the rate of men (Baker, 2002). Women’s decisions to interrupt their careers were difficult and were based on factors, such as workplace inflexibility, and their husbands’ lack of domestic responsibilities, rather than a preference to stay at home with their children (Stone and Lovejoy, 2004). Thus, both factors inside and outside the workplace constrain and shape women’s career decisions.

Our model is derived largely from research that has been conducted in male-dominated organizations; however, we speculate that it should hold for female-dominated organizations. There is evidence that tokenism does not work against men in terms of their promotion potential in female-dominated environments. Rather, there is some evidence for a glass-escalator effect for men in female-dominated fields, such as nursing, and social work (Williams, 1992). In addition, regardless of the gender composition of the workplace, men are advantaged, compared with women in terms of earnings and wage growth (Budig, 2002). Finally, even in female-dominated professions, segregation along gender lines occurs in organizational structure (Snyder and Green, 2008). Thus, the literature suggests that our model should hold for female-dominated environments.

Some might question if our model assumes that organizational decision makers enacting HR practices are men. It does not. There is evidence that decision makers who are women also discriminate against women (e.g., the Queen Bee phenomenon; Ellemers et al., 2004). Further, although men are higher in hostile sexism, compared with women (Glick et al., 1997, 2000), they are not necessarily higher in benevolent sexism (Glick et al., 2000). More importantly, the effects of hostile and benevolent sexism are not moderated by participant gender (Masser and Abrams, 2004; Salvaggio et al., 2009; Good and Rudman, 2010). Thus, those who are higher in hostile or benevolent sexism respond in a more discriminatory manner, regardless of whether they are men or women. Thus, organizational decision makers, regardless of their sex, should discriminate more against women in HR practices when they are higher in hostile or benevolent sexism.

In future work, the consequences of our model for women discriminated against in HR practices should be considered. The negative ramifications of sexism and discrimination on women are well known: physical and psychological stress, worse physical health (e.g., high blood pressure, ulcers, anxiety, depression; Goldenhar et al., 1998); lower job satisfaction, organizational commitment, and attachment to work (Murrell et al., 1995; Hicks-Clarke and Iles, 2000); lower feelings of power and prestige (Gutek et al., 1996); and performance decrements through stereotype threat (Spencer et al., 1999). However, how might these processes differ depending on the proximal cause of the discrimination?

Our model lays out two potential paths by which women might be discriminated against in HR practices: institutional discrimination stemming from organizational structures, processes, and practices and personal discrimination stemming from organizational decision makers’ levels of sexism. In order for the potential stressor of stigmatization to lead to psychological and physical stress it must be seen as harmful and self-relevant (Son Hing, 2012). Thus, if institutional discrimination in organizational structures, processes, and practices are completely hidden then discrimination might not cause stress reactions associated with stigmatization because it may be too difficult for women to detect (Crosby et al., 1986; Major, 1994), and label as discrimination (Crosby, 1984; Stangor et al., 2003). In contrast, women should be adversely affected by stigmatization in instances where gender discrimination in organizational structures, processes, and practices is more evident. For instance, greater perceptions of discrimination are associated with lower self-esteem in longitudinal studies (Schmitt et al., 2014).

It might appear that we have created a model, which is a closed system, with no opportunities to change an organization’s trajectory: more unequal organizations will become more hierarchical, and more equal organizations will become more egalitarian. We do not believe this to be true. One potential impetus for organizations to become more egalitarian may be some great shock such as sex-based discrimination lawsuits that the organization either faces directly or sees its competitors suffer. Large corporations have been forced to settle claims of gender harassment and gender discrimination with payouts upward of $21 million (Gilbert v. DaimlerChrysler Corp., 2004; LexisNexis, 2010; Velez, et al. v. Novartis Pharmaceuticals Crop, et al., 2010). Discrimination lawsuits are time consuming and costly (James and Wooten, 2006), resulting in lower shares, lower public perceptions, higher absenteeism, and higher turnover (Wright et al., 1995). Expensive lawsuits experienced either directly or indirectly should act as a big driver in the need for change.

Furthermore, individual women can work to avoid stigmatization. Women in the workplace are not simply passive targets of stereotyping processes. People belonging to stigmatized groups can engage in a variety of anti-stigmatization techniques, but their response options are constrained by the cultural repertoires available to them (Lamont and Mizrachi, 2012). In other words, an organization’s culture will provide its members with a collective imaginary for how to behave. For instance, it might be unimaginable for a woman to file a complaint of sexual harassment if she knows that complaints are never taken seriously. Individuals do negotiate stigmatization processes; however, this is more likely when stigmatization is perceived as illegitimate and when they have the resources to do so (Major and Schmader, 2001). Thus, at an individual level, people engage in strategies to fight being discriminated against but these strategies are likely more constrained for those who are most stigmatized.

Finally, possibly the most efficacious way for organizational members (men and women) to challenge group-based inequality and to improve the status of women as a whole is to engage in collective action (e.g., participate in unions, sign petitions, organize social movements, recruit others to join a movement; Klandermans, 1997; Wright and Lubensky, 2009). People are most likely to engage in collective action when they perceive group differences as underserved or illegitimate (Wright, 2001). Such a sense of relative deprivation involves feelings of injustice and anger that prompt a desire for wide scale change (van Zomeren et al., 2008). Interestingly, people are more likely to experience relative deprivation when inequalities have begun to be lessened, and thus their legitimacy questioned (Crosby, 1984; Kawakami and Dion, 1993; Stangor et al., 2003). If organizational leaders respond to such demands for change by altering previously gender oppressive organizational structures, processes, and practices, this can, in people’s minds, open the door for additional changes. Therefore, changes to mitigate gender inequalities within any organizational structure, policy, or practice could start a cascade of transformations leading to a more equal organization for men and women.

## Conflict of Interest Statement

The authors declare that the research was conducted in the absence of any commercial or financial relationships that could be construed as a potential conflict of interest.

## Footnotes

1 In this study, candidates were identified with initials and participants were asked to indicate the presumed gender of the candidate after evaluating them.
